# Phylogeography and the Evolutionary History of Sunflower (*Helianthus annuus* L.): Wild Diversity and the Dynamics of Domestication

**DOI:** 10.3390/genes11030266

**Published:** 2020-02-29

**Authors:** Brian Park, John M. Burke

**Affiliations:** Department of Plant Biology, University of Georgia, Miller Plant Sciences Bldg., Athens, GA 30602, USA; jmburke@uga.edu

**Keywords:** sunflower, phylogeography, domestication, demographic history, GBS

## Abstract

Patterns of genetic variation in crops are the result of selection and demographic changes that occurred during their domestication and improvement. In many cases, we have an incomplete picture of the origin of crops in the context of their wild progenitors, particularly with regard to the processes producing observed levels of standing genetic variation. Here, we analyzed sequence diversity in cultivated sunflower (*Helianthus annuus* L.) and its wild progenitor (common sunflower, also *H. annuus*) to reconstruct phylogeographic relationships and population genetic/demographic patterns across sunflower. In common sunflower, south-north patterns in the distribution of nucleotide diversity and lineage splitting indicate a history of rapid postglacial range expansion from southern refugia. Cultivated sunflower accessions formed a clade, nested among wild populations from the Great Plains, confirming a single domestication event in central North America. Furthermore, cultivated accessions sorted by market type (i.e., oilseed vs. confectionery) rather than breeding pool, recapitulating the secondary development of oil-rich cultivars during its breeding history. Across sunflower, estimates of nucleotide diversity and effective population sizes suggest that cultivated sunflower underwent significant population bottlenecks following its establishment ~5000 years ago. The patterns inferred here corroborate those from previous studies of sunflower domestication, and provide a comprehensive overview of its evolutionary history.

## 1. Introduction

Patterns of genetic variation in cultivated plants are the product of multiple processes that have occurred over their evolutionary histories. Gaining a comprehensive understanding of these patterns and the underlying processes requires reconstructing them within the broader context of the wild species from which they are derived. Such species-wide assessments can provide inferences into the ancestral lineages that gave rise to early domesticates and modern cultivars, and yield insights into the factors explaining the distribution of genetic diversity across gene pools. Such knowledge also has practical value, and can be applied to identify sources of novel alleles that should be preserved in germplasm collections and which may be of value in modern breeding programs [1,2,3,4]. Here, we characterize patterns of genetic variation in cultivated sunflower—a globally important oilseed crop—and its wild progenitor, common sunflower (both *Helianthus annuus* L.).

Common sunflower is a widely distributed annual herb whose native geographic range is centered in the Great Plains region of the United States and Canada [5]. Sunflower is thought to have been domesticated 3000–5000 years ago [6,7] by Native Americans who primarily used it as a source of edible seed [8]. Descendants of these early domesticates—the Native American landraces—were introduced to Europe in the early 16th century, and eventually made their way to Russia [9], where the predecessors of modern oilseed cultivars were developed and grown at an industrial scale [10]. These Russian oilseed cultivars were reintroduced to North America in the mid-20th century, stimulating commercial sunflower production in the Americas, and ushering in the modern era of sunflower breeding. More recent breeding efforts have transitioned sunflower from primarily open-pollinated varieties (OPVs) into a hybrid crop comprising two major market classes (i.e., oilseed and confectionery) that are separated into two major heterotic groups: unbranched, female (i.e., male-sterile; HA) lines and recessively branched, male restorer (i.e., RHA) lines [11,12].

This historical account of the evolutionary history of sunflower serves as the basis of hypotheses concerning the expected population structure and levels of standing genetic variation across its gene pool. Previous studies have sought to infer these patterns in sunflower at various scales, and have shed light on certain aspects of its origin and subsequent evolution. For example, broad examinations across common and cultivated sunflower have provided strong evidence for a single domestication event [13,14,15], and indicate that there was a marked reduction in genetic variation that corresponds to a domestication bottleneck [16,17,18,19,20,21]. More detailed examinations within cultivated sunflower have shown that there was a secondary loss of variation following improvement, and that cultivars are more or less genetically differentiated by market type (i.e., oilseed and confectionery; [16,17]) and breeding pool (e.g., RHA vs. HA; [21,22]). These studies have provided valuable insight into the genetic consequences of domestication and improvement in sunflower.

To date, however, attempts to infer patterns of population divergence and population structure in common sunflower (particularly from a phylogeographic perspective) have been somewhat limited, and efforts to formally reconstruct the demographic history of sunflower domestication are lacking. As such, a number of important issues have not been addressed. For example, given that common sunflower populations currently span areas with widely varying climatic conditions during the Last Glacial Maximum (LGM; ca. 21,500 ybp; [23,24,25]), phylogeographic analyses across the latitudinal range of the species may provide insights into: the locations of refugial areas where it may have survived the LGM, and how it colonized its current distribution following glacial retreat. Furthermore, given the differences in levels of genetic diversity between cultivated sunflower and its wild progenitor, demographic reconstructions would be useful for determining the timing and sequence of demographic changes that may explain observed patterns of variation across gene pools.

In this study, we sought to characterize the phylogeographic history of sunflower. We used genotyping-by-sequencing (GBS) to examine a panel of accessions that span the latitudinal range of common sunflower and the breadth of cultivated sunflower diversity to: (1) reconstruct the postglacial migration history of common sunflower; (2) identify patterns of genetic relatedness and structure across the various breeding pools of cultivated sunflower; and (3) gain insights into the history of demographic changes associated with domestication and subsequent improvement.

## 2. Materials and Methods

### 2.1. Sampling and Sequencing

**Figure 1:**
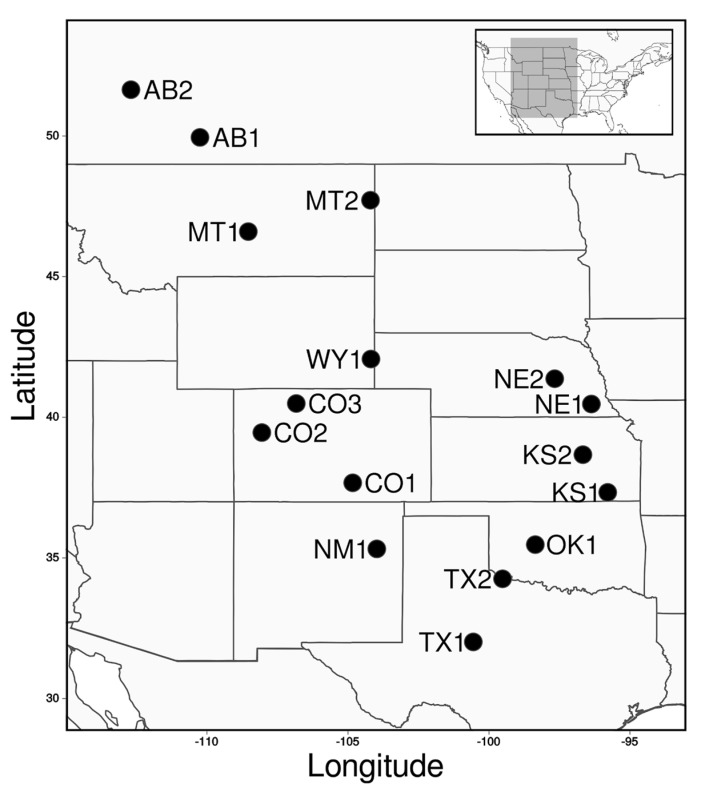
Sampling localities of the 16 populations of wild sunflower examined in this study.

We obtained seeds of cultivated and common sunflower from the USDA North Central Regional Plant Introduction Station (Ames, IA, USA; Table 1). Thirty cultivated accessions were selected to represent each of the major types of cultivated sunflower (twelve “exotic” lines, including six Native American landraces and six open-pollinated varieties [OPV]; eighteen “elite” lines including ten HA and eight RHA lines). Sixteen accessions of common sunflower (hereafter referred to as wild sunflower) were selected, to provide full latitudinal coverage of the central portion of its native range in the United States (Figure 1). Note that these populations were selected so as to avoid the potential for recent crop-wild contact, which would negatively impact our interpretation of observed patterns of variation. We also sampled individuals from one accession for each of two related wild species (*Helianthus argophyllus* and *Helianthus petiolaris*), to root phylogenies and polarize SNPs for the downstream analysis (see below). Seeds were sown in flats, and leaves were sampled from a single individual per cultivated sunflower line and up to 18 seedlings per wild sunflower accession. Genomic DNA was extracted from collected leaves using a modified CTAB protocol [26]. Resulting DNA extractions were checked for integrity on a 0.8% agarose gel and DNA quantity was measured with a Qubit 1.0 fluorometer (Invitrogen, Waltham, MA, USA).

DNA extractions were prepared for sequencing following a two-enzyme GBS protocol [27] using the restriction enzymes *Hpa*II and *Mse*I. Resulting libraries were pooled at 96-plex and sequenced on the Illumina Nextseq 500 sequencing platform (Illumina, San Diego, CA, USA) in high-output mode and set to produce 75 bp single-end reads. All library preparation and sequencing was performed at the Georgia Genomics and Bioinformatics Core (Athens, GA, USA).

### 2.2. Sequence Processing and Variant Calling

We used iPyrad version 0.9.16 [28] to process reads and call variants for downstream analysis. Briefly, raw demultiplexed reads were filtered with cutadapt 1.12 [29], to remove reads containing adapter sequences and > 5% low quality (phred score < 20) or ambiguous bases. Filtered reads were aligned to the HA412-HOv2 genome assembly [30] with BWA-MEM 0.7.17 [31] using default parameters, then sorted and indexed using samtools 1.10 [32]. Indexed reads were merged with BEDtools 2.29 [33], and bases were called for sites with ≥ 6 and ≤ 10,000 reads. Merged reads were clustered across samples and aligned into GBS loci, and loci with > 20% shared heterozygous sites and > 10% variable sites were filtered to remove poorly aligned and paralogous loci. Remaining loci anchored to the 17 chromosome-level scaffolds on the HA412-HOv2 genome assembly were retained, and SNP filtering was conducted in VCFtools 0.1.16 [34]. 

Phylogenetic analyses were conducted on the “phylogenomics” dataset composed of biallelic SNPs present in ≥ 50% of ingroup and outgroup samples (232 *H. annuus*; 2 *H. argophyllus,* 3 *H. petiolaris* samples) at a minor allele frequency greater than 1% (MAF > 0.01). The sample coverage threshold in this dataset was chosen because it allowed for the retention of lower coverage, high mutation rate sites which are useful in resolving recent divergences [35,36]. Population genetic analyses were conducted on three datasets, each consisting of both cultivated and wild sunflower samples (“ingroup_all”), and only wild (“ingroup_wild”) or cultivated (“ingroup_cultivated”) samples. The three datasets were composed of biallalic SNPs present in ≥ 80% of samples at MAF > 0.01, thinned to include SNPs that were ≥ 1 kb apart to reduce non-independence amongst sites. Demographic reconstructions were conducted on an “ingroup_dadi” dataset consisting of two samples per wild population (32 total) and all 30 of the cultivated samples. The “ingroup_dadi” dataset consisted of biallelic SNPs present in ≥ 50% of samples with a minor allele count of 2 (MAC = 2), spaced ≥ 1 kb apart, and polarized by alleles fixed in *H. petiolaris* and *argophyllus* (i.e., the ancestral state at each site was set to the alleles observed in *H. petiolaris* and *argophyllus*). The reduced sample size and sample coverage thresholds of the “ingroup_dadi” dataset were chosen as they increased the number of segregating sites available for demographic analysis. Furthermore, the relatively low MAF and MAC thresholds of the “ingroup” datasets were chosen to allow for the inclusion of rare variants which provide greater resolution of genetic structure and demographic events [37], while excluding singleton alleles that may reflect sequencing error.

### 2.3. Patterns of Genetic Diversity across Breeding Pools and Geographic Space

Estimates of mean nucleotide diversity (π) across all sites were calculated with VCFtools to identify patterns in the distribution of genetic diversity across breeding pools within *H. annuus* (i.e., wild, OPV, RHA, HA), as well as the wild populations, separately. Differences in π among breeding pools were assessed by computing the 95% confidence intervals from 1000 bootstrap replicates of per-site estimates of π using the “boot” and “boot.ci” functions in the R [38] package *boot* [39].

We then estimated patterns of genetic diversity across wild sunflower populations. Global population genetic parameters (e.g., Weir and Cockerham’s [40] F_ST_ and F_IS_) were estimated using all sites with the *hierfstat* R package [41]. Evidence of isolation-by-distance (IBD) was evaluated using a Mantel’s test with the “mantel.randtest” function as implemented in the R package *ade4* [42,43]. Pairwise estimates of Weir and Cockerham’s F_ST_ [40] were calculated using the “pariwise.WCfst” function in the *hierfstat* R package [41], and geographic distances between populations were calculated using the “distm” function in the *geosphere* R package [44]. Significance was assessed with 1 x 10^6^ Monte Carlo simulations. Clinal trends in π were investigated with linear regression, treating latitude and longitude as fixed effects using the “lm” function in R. AMOVA was conducted using the *poppR* package in R [45] to determine how genetic diversity is distributed across the following scales: between genetic clusters identified through fastSTRUCTURE (described below); among populations within genetic clusters; among samples within each population; and within samples. Significance was assessed with 1000 Monte Carlo simulations. 

### 2.4. Phylogenetic Relationships

We inferred phylogenetic relationships across wild and cultivated sunflower using RAxML 8.2.1 [46]. Analyses were conducted under GTR + CAT with ascertainment bias correction, with 20 tree searches and 100 bootstrap replicates to assess support. Trees were rooted with samples of *H. petiolaris*.

### 2.5. Population Clustering

We estimated individual ancestry coefficients with fastSTRUCTURE 1.0 [47] and ADMIXTURE 1.3 [48]. fastSTRUCTURE and ADMIXTURE analyses were run 5 times for *K* = 1–20 clusters using default parameters. The optimal number of clusters was determined using the “chooseK” tool and 10-fold cross-validation for fastSTRUCTURE and ADMIXTURE analyses, respectively. We then visualized samples in two-dimensional genetic space with principal component analysis (PCA) as implemented in the R package LEA [49].

### 2.6. Demographic History of Domestication

We modeled the divergence history between wild and cultivated sunflower using the diffusion approximation approach implemented in δaδi 2.0.3 [50]. We formulated three models that vary with respect to the presence and directionality of gene flow. Model A (Figure S1A) describes a simple divergence without gene flow scenario, where ancestral populations of sunflower split at time T into wild and cultivated lineages. Following the split, the wild lineage undergoes an instantaneous size change to a current effective size of Nwild-current, while the cultivated lineage has a founding effective size of Ncult-founder, that grows or declines to a current effective size, Ncult-current. Model B (Figure S1B) expands on Model A, and describes a divergence with gene flow scenario, allowing for symmetrical gene flow (Mw⟷c) between the wild and crop lineages. Similarly, Model C (Figure S1C) describes the same scenario as Model B, but allows for asymmetric gene flow (Mw→c, migration from wild into cultivated; Mw←c migration from cultivated into wild) between the lineages.

An unfolded 2D site-frequency spectrum was generated using the program *easySFS* (https://github.com/isaacovercast/easySFS), sampling 24 and 30 haplotypes from the cultivated and wild lineages, respectively, to maximize the number of segregating SNPs for analysis [50]. Model fitting was performed using *dadi_pipeline* (https://github.com/dportik/dadi_pipeline), described in Portik et al. [51]. *dadi_pipeline* was run using custom settings (rounds = 4; replicates = 50, 50, 50, 100; algorithm steps = 3, 5, 15, 50; -fold parameters = 3, 2, 2, 1) and models were extrapolated to a grid size of 40, 50, 60 points and fitted with Nelder-Mead optimization. Maximum-likelihood parameter estimates from the best replicate run (i.e., highest log-likelihood) for each model were used to calculate the Akaike information criterion (AIC) scores for model testing [52] following Carstens et al. [53]. Standard deviations for parameter estimates were obtained using the FIM approach [54], which has been demonstrated to provide reasonable uncertainty estimates for datasets composed of effectively unlinked SNPs, compared to more computationally expensive bootstrapping. Parameter estimates and their associated 95% confidence intervals were converted to biological units assuming a mutation rate of 6.1 × 10^−9^ substitutions/site/generation [55], and an effective sequence length ([bases sequenced to derive SNPs]*[SNPs used in the frequency spectrum/total number of SNPs called]) of L = 11.7 × 10^6^ bp.

## 3. Results

### 3.1. Sample Sizes and SNP Datasets

1.53 × 10^9^ reads were sequenced across 257 samples (222 wild, 30 cultivated, and 5 outgroup samples); on average, 5.96 × 10^6^ reads were sequenced per sample (range = 3.27 × 10^6^–1.47 × 10^7^ reads). Following quality filtering and processing, we assembled 1.08 × 10^6^ loci; the number of SNPs recovered from these loci are listed in Table 2.

### 3.2. Patterns of Genetic Diversity across Breeding Pools and over Geographic Space 

**Figure 2:**
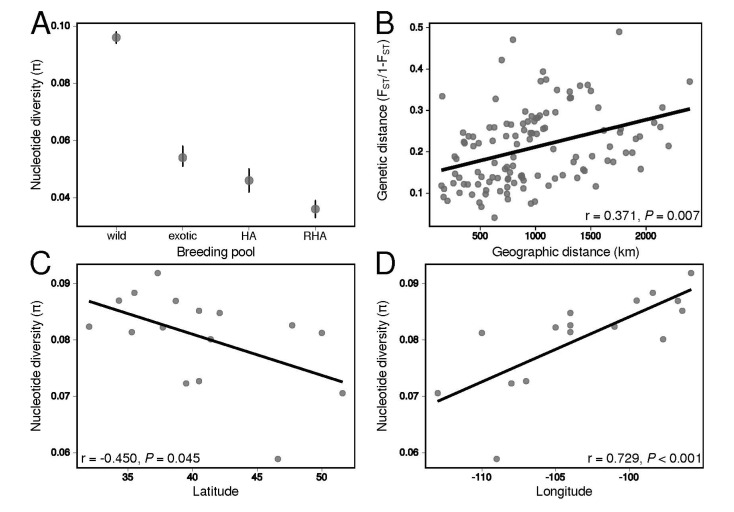
Patterns in the distribution of genetic diversity across breeding pools and over the geographic range of *H. annuus*. (**A**) Nucleotide diversity (π) varies significantly across breeding pools. In wild sunflower, pairwise genetic distances increase with geographic distances between populations (**B**). Furthermore, in wild sunflower, nucleotide diversity decreases with increasing latitude (**C**) and decreasing longitude (**D**).

There were notable differences in nucleotide diversity (π) among breeding pools (Figure 2A), with a roughly two-fold difference in π between wild sunflower and both the exotic and elite lines (mean (95% CI): wild sunflower = 0.096 (0.094–0.098); exotic = 0.054 (0.051–0.058); HA = 0.046 (0.042–0.049); RHA = 0.036 (0.033–0.039)). Differences in π amongst cultivated samples were less pronounced, but the HA and RHA lines possessed 85% and 66% of the nucleotide diversity present in the exotic lines, respectively. The marked differences in diversity across breeding pools in *H. annuus* indicates that primitive domesticated lines and improved cultivars harbor progressively less genetic diversity than their wild progenitor.

We observed moderate genetic differentiation (F_ST_ = 0.169) and inbreeding (F_IS_ = 0.177) across and within populations of wild sunflower. F_ST_ between populations varied widely (range = 0.048–0.336; Table S1), and genetic differentiation was found to be spatially structured, as indicated by a significant pattern of IBD (Figure 2B; Mantel’s *r* = 0.371, *P* = 0.007). Furthermore, clinal patterns in the distribution of diversity were observed, as indicated by significant declines in π with increasing latitude (Figure 2C; *F*_1,14_ = 4.82, *P* = 0.045, r = -0.450) and decreasing longitude (Figure 2D; *F*_1,14_ = 18.1, *P* < 0.001, r = 0.729). AMOVA found that most genetic variation is partitioned within samples (68.8%, *P* < 0.001), with relatively little variation explained by differences between samples (13.2%, *P* < 0.001), populations (13.3%, *P* < 0.001), and genetic clusters (4.56%, *P* < 0.001). The sum of these results suggest that genetic diversity is continuously distributed across the range of wild sunflower, with the highest levels of diversity being concentrated in populations located in the southeastern portion of the range.

### 3.3. Phylogenetic Relationships

**Figure 3:**
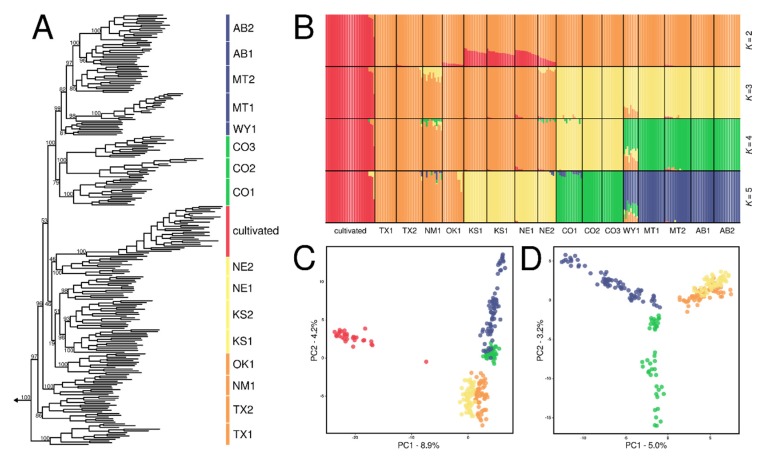
Phylogenetic relationships and population structure across sunflower. (**A**) Maximum likelihood phylogeny of wild and cultivated sunflower. ML BS support values are denoted for interior nodes and clades corresponding to populations. (**B**) Population assignments for *K* = 2-5 estimated through fastSTRUCTURE. (**C**) Positioning of wild and cultivated sunflower samples in two-dimensional genetic space along PCs 1 and 2. (**D**) Positioning of wild sunflower samples in two-dimensional genetic space along PCs 1 and 2. Colored bars in panel A and colored dots in panels C and D correspond to fastSTRUCTURE groups for *K* = 5 in panel B.

Phylogenetic analyses infer a clear geographic pattern of lineage splitting in wild sunflower (Figure 3A). Samples from each population were resolved as monophyletic (ML BS > 85), with the exception of the population in Wyoming (WY1), where one sample was resolved as sister to a clade of samples from a neighboring population in Montana. The earliest diverging lineage in our sample of wild sunflower was resolved as a population from south Texas (TX1; ML BS = 100), followed by a population from central Texas (TX2; ML BS = 97). Samples from outside of Texas form a well-supported clade (ML BS = 96), with populations from New Mexico and the central Great Plains region (Oklahoma, Kansas, and Nebraska) forming a grade (i.e., relationships resolved among these populations were resolved with ML BS < 50), with respect to a strongly-supported western clade (ML BS = 100), comprising populations from Colorado, Wyoming, Montana, and Alberta. Relationships in the western clade show a south-north pattern of lineage splitting, mirroring the patterns observed more broadly across wild sunflower. Taken together, these phylogenetic patterns suggest that range expansion in wild sunflower occurred along two separate, south–north migration fronts, with multiple genetic lineages colonizing the central portion of the Great Plains, and a single genetic lineage migrating into and diversifying over the western portion of its range.

**Figure 4:**
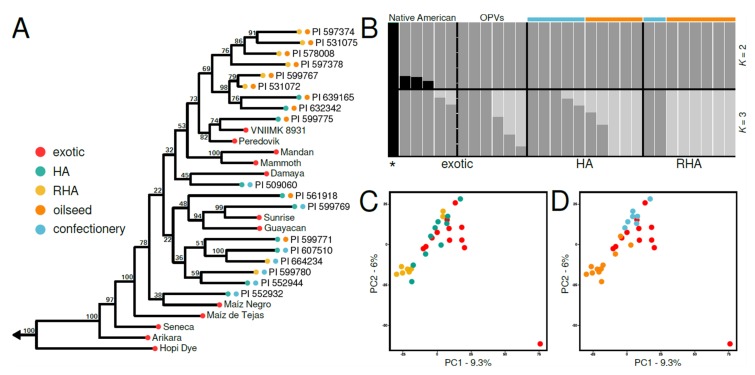
Phylogenetic relationships and population structure within cultivated sunflower. (**A**) Maximum likelihood phylogeny of cultivated accessions. Tip shapes are colored by breeding pool and market type (i.e., oilseed vs. confectionery), as shown in the inset. ML BS support values are noted for each node. (**B**) Population assignments for *K* = 2–3, estimated through fastSTRUCTURE. The dashed line demarcates Native American landraces (left) from OPVs (right). The asterisk denotes the Native American landrace accession, Hopi Dye. Colored bars denote market type for each accession follow the coding scheme shown in the inset of panel A. Positioning of cultivated samples in two-dimensional genetic space along PCs 1 and 2 coded by breeding pool (**C**) and market type (**D**).

All cultivated sunflower accessions were resolved as a strongly supported clade (ML BS = 100) nested within the New Mexico-Central Great Plains grade (Figure 3A). Four Native American landraces (i.e., Hopi Dye, Arikara, Seneca, Maíz de Tejas, Maíz Negro) diverged early and form a grade at the base of the cultivated clade (Figure 4A). There is little phylogenetic structure after the early diverging Native American landraces, but cultivated accessions appear to sort largely by market type (i.e., oilseed vs. confectionery) rather than heterotic group. This is apparent in the resolution of all but two of the oilseed lines as a clade (the most-inclusive clade containing the oilseed lines PI 599775 (HA123) and two high-oil OPVs (Peredovik and VNIIMK 8931)), and the paraphyly of the confectionery lines. Two oilseed lines (PI 599771 (HA061) and PI 561918 (HA378)) cluster with the confectionery lines, which may be a result of introgressions rather than independently derived oilseed lines. Overall, relationships among cultivated accessions are decidedly complex, but the resolution of Native American landraces at the base of the cultivated sunflower phylogeny is consistent with the view that all modern cultivars of sunflower are descended from Native American landraces. Furthermore, the paraphyly of the confectionery lines, and the sorting of most oilseed lines into a clade, indicate that the oilseed lines were derived from a non-oilseed progenitor, consistent with the known breeding history of cultivated sunflower.

### 3.4. Population Clustering

fastSTRUCTURE and ADMIXTURE analyses infer diffuse, geographically defined population structure across wild sunflower. Both analyses disagree with respect to the optimal value of *K* (fastSTRUCTURE, *K* = 3; ADMIXTURE, *K* = 8), but consistently identified a distinct cluster of cultivated accessions while sorting wild samples into increasingly smaller, geographically defined clusters until *K* = 17, where all wild samples were sorted by their collecting locality. This fractal pattern of population clustering is consistent with IBD, so we present results from fastSTRUCTURE analyses for *K* = 2–6, which circumscribe landscape-level, geogenetic clusters (Figure 3B). At *K* = 2, samples were sorted into cultivated and wild clusters. At *K* = 3, the wild samples split into two clusters corresponding to a southern/eastern and a western cluster. At *K* = 4, the western cluster split into a southern-western cluster composed of samples from Colorado populations and a northern-western cluster composed of samples from Wyoming, Montana, and Alberta. Finally, at *K* = 5, the southern-eastern cluster split into a southern cluster composed of samples from Texas, New Mexico and Oklahoma and an eastern cluster composed of samples from Kansas and Nebraska. Instances of admixture were uncommon across populations, with most samples possessing > 80% ancestry in a given cluster. However, one Native American landrace accession (Hopi Dye) was consistently estimated to have ca. 50% membership in other clusters across all values of *K*, and samples from the Wyoming population were found to possess 30-40% admixed ancestry at *K* = 4 and 5 (Figure 3B). PCA recapitulates these patterns, with cultivated accessions positioned distantly from wild samples (Figure 3C), and wild samples from nearby populations grouping together in PC space (Figure 3D). 

The population structure within cultivated sunflower is more complex. fastSTRUCTURE and ADMIXTURE analyses favored lower values of *K* (fastSTRUCTURE, *K* = 2; ADMIXTURE, *K* = 1) and, for both analyses, ancestry assignments at *K* > 3 were difficult to interpret. That being said, we present fastSTRUCTURE results for *K* = 2–3, which reveal subtle, biologically interpretable structure within the cultivated lines. At *K* = 2, a single Native American landrace accession (Hopi Dye) is inferred as its own cluster, with three other Native American landrace accessions (Arikara, Seneca, Maíz de Tejas) sharing some ancestry (< 20%) with the Hopi Dye cluster (Figure 4B). At *K* = 3, Hopi Dye remains a unique cluster, and two additional clusters emerge to separate accessions largely by market type (i.e., oilseed vs. confectionery lines) rather than breeding pool (Figure 4B). PCA also infers subtle structure, with little separation of accessions by breeding pool (Figure 4C), and some differentiation occurring among market types (Figure 4D).

### 3.5. Demographic Reconstruction

Demographic reconstructions estimate that the wild and cultivated sunflower lineages diverged between 900–5400 ybp (Table 3). All models estimate current effective size of the wild lineage (Nwild-current) to be roughly 10- to 20-times greater than the current effective size of the cultivated lineage (Ncult-current). Similarly, all models estimate up to a 20-fold reduction in effective size between the founding (Ncult-founder) and contemporaneous (Ncult-current) cultivated lineage. These dramatic differences in current and historical population sizes between the cultivated and wild lineages are consistent with significant losses of genetic diversity during domestication and subsequent improvement.

**Figure 5:**
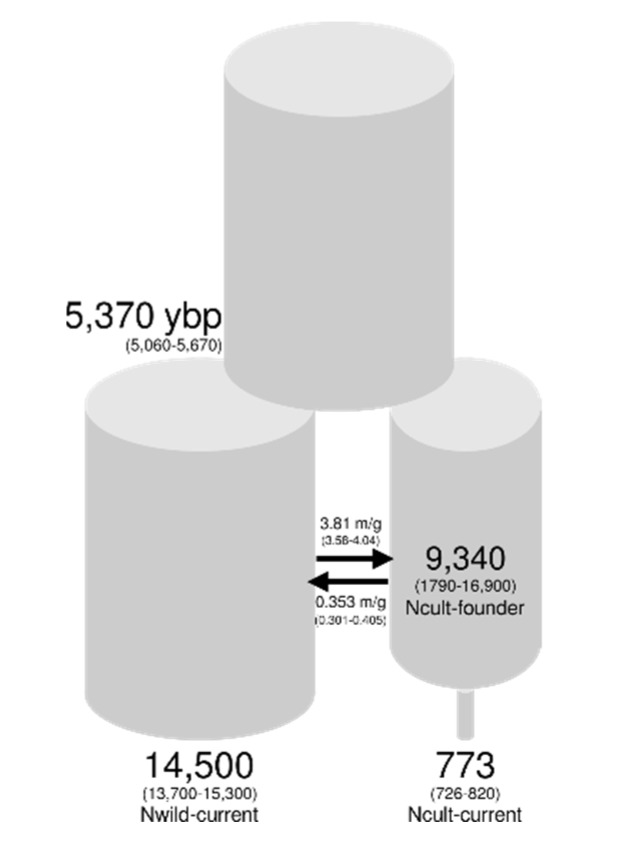
Parameter estimates and 95% confidence intervals (in parentheses) for a demographic model of sunflower domestication assuming divergence with asymmetric gene flow (Model C).

AIC favored model C (Table 3; Figure 5; Figure S2), which models asymmetric migration between the cultivated and wild lineages. In this model, the cultivated and wild lineages diverged 5370 ybp, with the cultivated lineage undergoing sequential bottlenecks, ultimately resulting in a nearly 20-fold reduction in the effective size of the modern breeding pool. Migration from the wild lineage into the cultivated lineage (Mw→c) was estimated at 3.81 migrants per year (95% CI: 3.58–4.04), which is an order of magnitude greater than migration from the cultivated into wild lineage (Mw←c = 0.35 (0.301–0.405)).

## 4. Discussion

### 4.1. Phylogeography of Wild Sunflower

Populations across the range of wild sunflower have diverged primarily along a south-north axis (Figure 3A), which is consistent with a scenario of postglacial range expansion from a southern refugium. The observed pattern of IBD (Figure 2B), partitioning of most genetic diversity at finer spatial scales, and relatively weak population structure (Figure 3B–D) indicates that genetic diversity is continuously distributed over the species range, and suggests that range expansion occurred rapidly following glacial retreat. Furthermore, linear declines in nucleotide diversity with increasing latitude and decreasing longitude (Figure 2C,D) indicate that range expansion likely occurred in a stepwise fashion [56], through sequential founding events as colonizing populations migrated north- and westward from refugial populations located in the southeastern portion of the range.

Together, these findings suggest that the dynamics of postglacial range expansion in wild sunflower are similar to those observed in numerous European plant species. Indeed, many such species have been found to have undergone dramatic range contractions into southern refugia during glacial periods and rapidly expanded northward as climates warmed following the LGM [23,24,25,57]. This general pattern of northward expansion from lower latitude refugial areas has also been observed in a number of widespread species, whose contemporary distributions span both glaciated and unglaciated North America: e.g., herbs (*Asclepias exaltata* [58], *Campanulastrum americanum* [59], *Trillium erectum* and *T. grandiflorum* [60,61], and *Symplocarpus foetidus* [62]); shrubs (*Dirca paulustris* [63], *Viburnum lantanoides* [64], *Viburnum nudum* complex [65], and the *Lentago* clade of *Viburnum* [66]); and trees (e.g., *Acer rubrum* and *sacharum* [67,68], *Carya cordiformis* and *ovata* [69,70], *Fagus grandifolia* [67,71], and *Pinus strobus* [72,73]).

Paleoecological studies provide a finer resolution to the approximate locations of refugial areas during the late Pleistocene, and suggest that many of the aforementioned species may have persisted in macrorefugia distributed along the Gulf Coast [74], with colder-adapted species surviving in smaller refugia along the Atlantic Coast [75], or in cryptic microrefugia at mid-latitudes [76,77]. In the case of wild sunflower, there are no fossils that place it in any of these regions during the LGM. However, there are records of composite pollen originating during the LGM from eastern Texas, the lower Mississippi River Valley, peninsular Florida, and the coastal Carolinas [74]. Given that the core of the wild sunflower distribution is centered in the Great Plains region [5], and that phylogenetic and genetic diversity is concentrated in the southern and eastern portions of its range, we postulate that its refugial areas may have been located in adjacent areas such as eastern Texas and the lower Mississippi River Valley, both of which harbored grassland species during the LGM [74]. Future ecogeographic studies incorporating well-curated distributional data of wild sunflower populations and paleoclimatic niche modelling would serve as excellent tests of this hypothesis.

Our analysis of wild sunflower adds to the growing body of work demonstrating the general trend of south-north postglacial migration inferred in wide-ranging North American plant species. Interestingly, there is a dearth of phylogeographic studies that have examined a wide-ranging plant species that spans the entirety of the Great Plains (but see [78]). As such, our study provides interesting insights into central North American plant phylogeography. One pattern inferred in wild sunflower is the resolution of separate south-north patterns of lineage splitting in the western and central Great Plains region (Figure 3A), with multiple genetic lineages colonizing the central Great Plains, and a single genetic lineage migrating and diversifying over the western Great Plains. This pattern may be explained by the physiography of the western Great Plains, which is at higher elevation and possesses a cool, arid climate. This region—the High Plains—has been shown to be an important biogeographic break for many animals in North America [79], where previously isolated taxa/populations situated on either side of the region have been shown to have come into secondary contact as climates warmed during the Holocene [80,81,82]. Indeed, in our study, samples from a population collected in eastern Wyoming were resolved as paraphyletic (Figure 3A), possessing slightly greater nucleotide diversity compared to other populations at similar latitudes (Figure 2C) and having > 30% admixed ancestry at higher values of *K* (Figure 3B). Our findings in wild sunflower suggest that the High Plains played an important role in generating contemporary patterns of divergence and genetic structure in not just animals, but wide-ranging plants distributed across central North America.

### 4.2. Insights into the Domestication and Breeding History of Cultivated Sunflower

Patterns of divergence and population structure in cultivated sunflower are complex, but largely reflective of its domestication and breeding history. Cultivated accessions form a strongly supported clade, nested among wild sunflower populations from the central Great Plains (Figure 3A), and estimated to have arisen into an independently evolving entity ca. 5370 ybp (Table 3; Figure 5). Within the cultivated clade, five Native American landraces (Hopi Dye, Arikara, Seneca, Maíz de Tejas, and Maíz Negro) were resolved as the earliest diverging lineages, which split in succession from a single founding lineage and eventually gave rise to the modern cultivars (Figure 4A). These findings agree well with those from previous studies, demonstrating that extant sunflower cultivars trace back to a single origin of domestication [13,14,15] ca. 5000 ybp [7] in east-central North America [13,16]. Propagules of this initial domestication were then presumably dispersed between different Native American cultures who used it for food and cultural purposes [8].

Patterns of phylogenetic and population structure outside of the early diverging landraces become apparent when the cultivars are coded by market type (i.e., oilseed vs. confectionery) rather than breeding pool (i.e., exotic vs. HA vs. RHA) (Figure 4). The sorting of cultivars by market type is not unexpected [17,21,22], as early breeding efforts in Eastern Europe were focused on increasing oil content [10], which likely resulted in substantial genetic differentiation. The development of inbred lines and accompanying transition to hybrid breeding occurred much more recently [83]. Our results reflect this history, where nearly all oilseed lines were resolved as a clade in relation to a grade composed of confectionery lines, with two Russian developed high-oil OPVs (Peredovik and VNIIMK 8931) splitting early within the oilseed clade’s history. Given the limited sample of accessions included in our study, future studies examining a greater number and diversity of both wild and domesticated lineages will be useful in confirming the patterns inferred in this study, and gaining more pointed insights into the origin of domesticated sunflower and the effects of historical breeding efforts in generating observed patterns of relatedness and genetic structure. Of particular value might be an expansion of the wild sunflower sampling to provide better coverage of the eastern and western portions of its range.

### 4.3. Domestication and Its Effects on Polymorphism 

Domestication and improvement have generated large differences in observed levels of genetic diversity in the wild and cultivated sunflower breeding pools. Unsurprisingly, exotic and elite lines were found to harbor ca. 60% and 50% of the nucleotide diversity (π) present in wild sunflower, respectively (Figure 2A). These results compare favorably with those from previous surveys of SSR and SNP diversity in sunflower, which have consistently estimated up to a 50% reduction in various measures of diversity (primarily gene diversity (H_e_)) between wild and cultivated sunflower [16,17,18,19,20,21,84]. The consistency across studies and marker types suggest that the effects of domestication and improvement were dramatic, affecting both SSR and SNP variation across the sunflower genome.

Demographic reconstructions provide some insight into the timing and order of these changes in genetic diversity (Table 3; Figure 5). For example, we inferred a dramatic 12-fold reduction in effective size over the history of the cultivated lineage (i.e., Ncult-founder vs. Ncult-current) and a more subtle 1.5-fold difference in effective sizes between the wild lineage and the founding population of the cultivated lineage (i.e., Nwild-current vs. Ncult-founder). These results support the notion that genetic diversity in cultivated sunflower was lost progressively, with a moderate loss of diversity during the initial domestication bottleneck, and more severe reductions in diversity following strong directional selection and additional bottlenecks during improvement (reviewed in [85,86,87]). Losses in genetic diversity following domestication and improvement are a common feature of many cultivated plant species [87,88]. However, the patterns observed in sunflower contrasts with those reconstructed in other annual crops such as common bean and maize, where current effective sizes are much larger than their inferred domestication bottleneck sizes, possibly due to rapid population expansion or ongoing gene flow with wild relatives [89,90,91].

In sunflower, moderate rates of gene flow from the wild into cultivated breeding pools do not appear to have strongly influenced current effective sizes (Table 3; Figure 5), which may be reflective of the targeted nature of introgression events in cultivated sunflower breeding (e.g., the introduction of disease resistance loci from wild donors [84,92,93]). Overall, these results are consistent with the known history of cultivated sunflower, but many issues remain unresolved: specifically, the duration of the domestication bottleneck, and the tempo and mode of bottleneck-induced population declines. A genomic analysis of contemporary and archeological specimens (e.g., [94]) with recently developed methods designed to infer more granular changes in effective population size through time [95] may be useful in generating richer insights into the broad demographic patterns observed in this study.

## Figures and Tables

**Table 1:** Accession numbers, improvement status, and geographic origins of wild and cultivated lines examined in this study. Cultivated accessions are categorized as exotic (i.e., Native American landraces and open-pollinated varieties [OPVs]), HA, or RHA lines. Market type (i.e., oilseed or confectionery) is denoted for HA/RHA lines. All seeds were obtained from the North Central Regional Plant Introduction Station (Ames, IA, USA).

USDA PI Number	Improvement Status	Name	Geographic Origin	Sample Size
592304	wild	AB2	Alberta, CAN; 51.6, -112.7	16
592309	wild	AB1	Alberta, CAN; 49.9, -110.2	14
586816	wild	MT2	Montana, USA; 47.7, -104.2	16
531035	wild	MT1	Montana, USA; 46.6, -108.5	16
586837	wild	WY1	Wyoming, USA; 42.07, -104.18	9
435564	wild	CO3	Colorado, USA; 40.49, -106.83	13
468622	wild	CO2	Colorado, USA; 39.45, -108.05	12
435560	wild	CO1	Colorado, USA; 37.67, -104.83	16
586869	wild	NE2	Nebraska, USA; 41.37, -97.67	11
586866	wild	NE1	Nebraska, USA; 40.47, -96.37	14
586859	wild	KS2	Kansas, USA; 38.67, -96.67	17
664770	wild	KS1	Kansas, USA; 37.33, -95.79	14
468489	wild	OK1	Oklahoma, USA; 35.47, -98.36	13
435479	wild	NM1	New Mexico, USA; 35.32, -103.98	12
435366	wild	TX2	Texas, USA; 34.26, -99.52	16
649848	wild	TX1	Texas, USA; 32.01, -100.55	13
607510	elite – HA, confectionery	HAR7	USDA Breeding Program	1
599780	elite – HA, confectionery	HA285	USDA Breeding Program	1
599769	elite – HA, confectionery	HA008	USDA Breeding Program	1
552932	elite – HA, confectionery	HA286	USDA Breeding Program	1
509060	elite – HA, confectionery	HA350	USDA Breeding Program	1
639165	elite – HA, oilseed	HA442	USDA Breeding Program	1
632342	elite – HA, oilseed	HA433	USDA Breeding Program	1
599775	elite – HA, oilseed	HA124	USDA Breeding Program	1
599771	elite – HA, oilseed	HA061	USDA Breeding Program	1
561918	elite – HA, oilseed	HA378	USDA Breeding Program	1
664234	elite – RHA, confectionery	RHA325	USDA Breeding Program	1
552944	elite – RHA, confectionery	RHA282	USDA Breeding Program	1
599767	elite – RHA, oilseed	RHA299	USDA Breeding Program	1
597378	elite – RHA, oilseed	RHA400	USDA Breeding Program	1
597374	elite – RHA, oilseed	RHA397	USDA Breeding Program	1
578008	elite – RHA, oilseed	RHA386	USDA Breeding Program	1
531075	elite – RHA, oilseed	RHA362	USDA Breeding Program	1
531072	elite – RHA, oilseed	RHA359	USDA Breeding Program	1
294659	exotic – OPV	Peredovik	Russia, Asia	1
340790	exotic – OPV	VNIIMK8931	Russia, Asia	1
476853	exotic – OPV	Mammoth	Russia, Asia	1
496263	exotic – OPV	Damaya	China, East Asia	1
162454	exotic – OPV	Sunrise	USA, North America	1
650353	exotic – OPV	Guayacan	Uruguay, South America	1
369357	exotic – Native American landrace	Arikara	USA, North America	1
369360	exotic – Native American landrace	Seneca	USA, North America	1
432504	exotic – Native American landrace	Hopi Dye	USA, North America	1
600717	exotic – Native American landrace	Mandan	USA, North America	1
650646	exotic – Native American landrace	Maíz Negro	USA, North America	1
650761	exotic – Native American landrace	Maíz de Tejas	USA, North America	1
435624	outgroup – wild *H. argophyllus*	-	Texas, USA; 28.17, -97.00	2
613764	outgroup – wild *H. petiolaris*	-	North Dakota, USA; 46.86, -96.90	3

**Table 2:** The number of SNPs present in each dataset analyzed in this study. See text for details regarding the composition of the individual datasets.

Dataset	Samples	Sample Coverage	MAF/MAC Threshold	Thinning Interval	SNPs
phylogenomics	257	50%	MAF = 0.01	-	43,271
ingroup_all	252	80%	MAF = 0.01	1 kb	5745
ingroup_wild	222	80%	MAF = 0.01	1 kb	5571
ingroup_crop	30	80%	MAF = 0.01	1 kb	12,808
ingroup_dadi	62	50%	MAC = 2	1 kb	12,025

**Table 3:** Maximum likelihood estimates for model parameters estimated using δaδi. 95% confidence intervals for parameter estimates are presented in parentheses. Migration rates (M) are presented in migrants per year.

Model	ll	AIC	ΔAIC	Nref (×10^3^)	N_wild-current_ (×10^3^)	N_cult-founder_ (×10^3^)	N_cult-current_ (×10^3^)	Mw⟷c	Mw→c	Mw←c	T Years (×10^3^)
A	−3303	6614	2719	8.51 (8.30–8.73)	14.7 (13.8–15.7)	8.75 (7.35–10.1)	0.865 (0.792–0.938)	-	-	-	0.912 (0.776–1.06)
B	−2123	4255	361	4.58 (4.31–4.86)	13.2 (12.2–14.1)	21.0 (10.8–31.3)	1.51 (1.39–1.63)	3.06 (2.96–3.16)	-	-	4.85 (4.32–5.37)
C	−1941	3895	0	4.23 (4.06–4.39)	14.5 (13.7–15.3)	9.34 (1.79–16.9)	0.773 (0.726–0.820)	-	3.81 (3.58–4.04)	0.353 (0.301–0.405)	5.37 (5.06–5.67)

## Supplementary Materials

The following are available online at https://www.mdpi.com/2073-4425/11/3/266/s1, Table S1: Pairwise F_ST_ estimates between the cultivated accessions and wild sunflower populations, Figure S1: Schematic representations of demographic models fitted using δaδi. Estimated parameters are noted with text for each model, Figure S2. δaδi analysis of model C. Upper panels are the observed and expected site frequency spectra. Lower panels are a heat map and histogram of residuals.

## Data Accessibility

Illumina sequence data generated in this study are accessible from the NCBI Sequence Read Archive (https://www.ncbi.nlm.nih.gov/sra) under BioProject number PRJNA609331.
