# Supplementary material for: Phylogeography and the Evolutionary History of Sunflower (Helianthus annuus L.): Wild Diversity and the Dynamics of Domestication
Source: Genes (Basel). 2020 Feb 29;11(3):266. doi: 10.3390/genes11030266 (PMC7140811; doi:10.3390/genes11030266)
Supplement: Supplementary file 1 [file genes-11-00266-s001.pdf]

Supplementary Material: Phylogeography and the Evolutionary History of Sunflower (*Helianthus annuus* L.): Wild Diversity and the Dynamics of Domestication

Brian Park and John M. Burke

Table S1. Pairwise F_{ST} estimates between the cultivated accessions and common sunflower populations.

	**Cult.**	**TX1**	**TX2**	**NM1**	**OK1**	**KS1**	**KS2**	**NE1**	**NE2**	**CO1**	**CO2**	**CO3**	**WY1**	**MT1**	**MT1**	**AB1**
TX1	0.423															
TX2	0.339	0.158														
NM1	0.363	0.179	0.091													
OK1	0.352	0.193	0.094	0.124												
KS1	0.335	0.193	0.107	0.134	0.130											
KS2	0.317	0.194	0.097	0.121	0.114	0.081										
NE1	0.320	0.194	0.100	0.121	0.113	0.106	0.075									
NE2	0.340	0.208	0.110	0.124	0.118	0.128	0.095	0.099								
CO1	0.364	0.214	0.123	0.129	0.153	0.160	0.142	0.139	0.143							
CO2	0.443	0.296	0.204	0.218	0.229	0.231	0.220	0.220	0.227	0.212						
CO3	0.433	0.284	0.196	0.212	0.223	0.232	0.216	0.212	0.218	0.195	0.263					
WY1	0.344	0.171	0.073	0.081	0.104	0.119	0.097	0.090	0.090	0.079	0.184	0.165				
MT1	0.460	0.336	0.248	0.264	0.267	0.277	0.264	0.265	0.269	0.248	0.335	0.319	0.200			
MT2	0.346	0.211	0.111	0.126	0.136	0.149	0.120	0.120	0.118	0.113	0.207	0.190	0.048	0.201		
AB1	0.374	0.232	0.135	0.148	0.161	0.162	0.148	0.148	0.145	0.137	0.231	0.211	0.072	0.206	0.066	
AB2	0.405	0.278	0.182	0.200	0.208	0.216	0.189	0.194	0.196	0.183	0.277	0.259	0.126	0.259	0.123	0.110

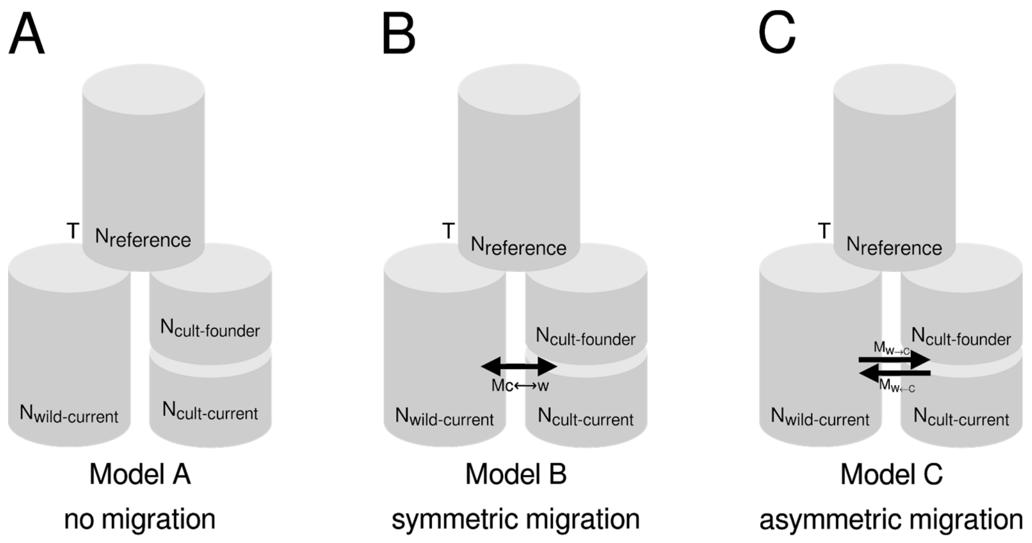

Figure S1. Schematic representations of demographic models fitted using ∂a∂i. Estimated parameters are noted with text for each model.

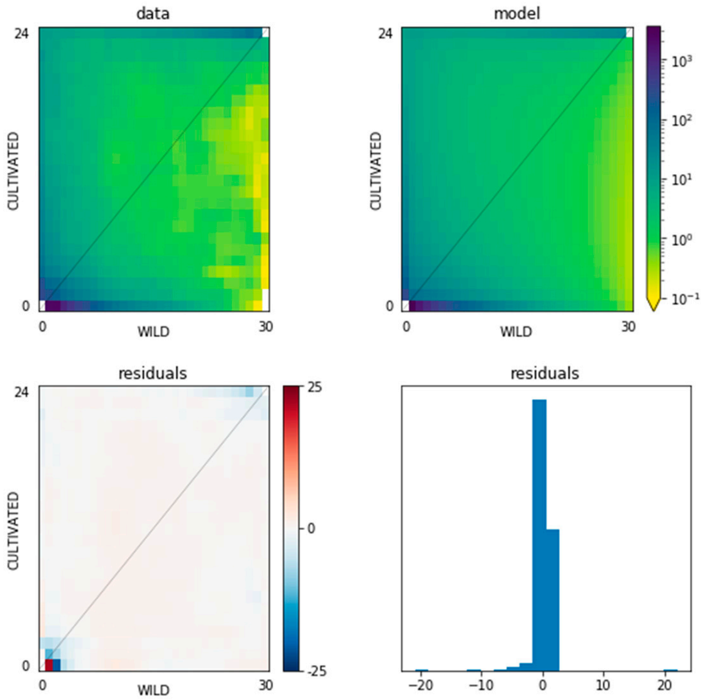

Figure S2. ∂a∂i analysis of model C. Upper panels are the observed and expected site frequency spectra. Lower panels are a heat map and histogram of residuals.
